# Ginsenoside RG1 enhances the paracrine effects of bone marrow-derived mesenchymal stem cells on radiation induced intestinal injury

**DOI:** 10.18632/aging.202241

**Published:** 2020-12-03

**Authors:** Yujun Luo, Beibei Wang, Jianhua Liu, Faxin Ma, Dongling Luo, Zhongwen Zheng, Quan Lu, Weijie Zhou, Yue Zheng, Chen Zhang, Qiyi Wang, Weihong Sha, Hao Chen

**Affiliations:** 1 Shantou University Medical College, Shantou 515041, Guangdong, P.R. China; 2 The Second School of Clinical Medicine, Southern Medical University, Guangzhou 510515, Guangdong, P.R. China; 3 Department of Gastroenterology, Guangdong Provincial People’s Hospital, Guangdong Academy of Medical Sciences, Guangzhou 510080, Guangdong, P.R. China; 4 Department of Oncology, Cancer Center, Guangdong Provincial People’s Hospital, Guangdong Academy of Medical Sciences, Guangzhou 510080, Guangdong, P.R. China; 5 Department of Gastroenterology, Shantou Central Hospital, Affiliated Shantou Hospital of Sun Yat-sen University, Shantou 515041, Guangdong, P.R. China; 6 Department of Cardiology, Guangdong Cardiovascular Institute, Guangdong Provincial People’s Hospital, Guangdong Academy of Medical Sciences, Guangzhou 510080, Guangdong, P.R. China

**Keywords:** mesenchymal stem cells, conditioned medium, heme oxygenase-1, ginsenoside RG1, radiation induced intestinal injury

## Abstract

Content and aims: Ginsenoside RG1 (RG1) is thought to enhance proliferation and differentiation of stem cell, however, its role on paracrine efficacy of stem cell remains unclear. Here we examined if and how RG1 enhances the paracrine effects of bone marrow-derived mesenchymal stem cells (BM-MSCs) on radiation induced intestinal injury (RIII).

Method: Irradiated rats randomly received intraperitoneal injection of conditioned medium (CM) derived from non-activated BM-MSCs (MSC-CM) or BM-MSCs pre-activated by RG-1 (RG1-MSC-CM). Intestinal samples were collected, followed by the evaluation of histological and functional change, apoptosis, proliferation, inflammation, angiogenesis and stem cell regeneration. The effects of heme oxygenase-1 (HO-1) were investigated using HO-1 inhibitor or siRNA.

Result: RG1 enhanced the paracrine efficacy of BM-MSCs partially through upregulation of HO-1. RG1-MSC-CM rather than MSC-CM significantly improved the survival and intestinal damage of irradiated rats via improvement of intestinal proliferation/apoptosis, inflammation, angiogenesis and stem cell regeneration in a HO-1 dependent mechanism. The mechanism for the superior paracrine efficacy of RG1-MSC-CM is related to a higher release of two pivotal cytokines VEGF and IL-6.

Conclusion: Our study revealed that RG1 enhances paracrine effects of BM-MSCs on RIII, providing a novel method for maximizing the paracrine potential of MSCs.

## INTRODUCTION

Radiation induced intestinal injury (RIII) is common among patients with intra-abdominal and/or pelvic malignancy. The radiation damage caused by radiotherapy triggers epithelial apoptosis or eventual necrosis in the intestine [1, 2]. It has been reported that 3~10% of patients develop a severe form of radiation-induced bowel injury such as intestinal strictures/fistula or even enterogenous sepsis thus affecting their quality of life [2]. Despite these challenges, there are still no effective strategies in treating this life-threatening disease.

Stem cell therapy using mesenchymal stem cells (MSCs) has been fronted as a promising therapy for treating the adverse effects related to radiotherapy [1, 3]. Although earlier studies attributed the therapeutic effect of MSCs to the ability to migrate and implant into damaged tissues, it has now been considered that the secretome of MSCs is primarily responsible for the observed benefits. MSCs can secret numerous trophic molecules such as growth factors as well as extracellular vesicles in a paracrine or autocrine manner, which enhances the regeneration of the host cells [4–6]. Though transplanted MSCs have beneficial effect on tissue regeneration, MSCs transplantation is hampered by low rate of transplantation and a risk of tumorigenesis [7–9]. MSC-derived conditioned medium (MSC-CM) containing numerous trophic factors represents an alternative to MSC transplantation [10]. It has been found that MSC-CM can exert its protective effect on tissue regeneration by inhibiting cellular apoptosis, reducing inflammation, promoting proliferation and improving neoangiogenesis without the risks of tumorigenesis and immune rejection [4, 5, 7, 8].

Despite the beneficial effects of MSC-CM being reported by many studies, its application is limited by low concentration of trophic factors [11, 12]. For example, it has been reported that the minimal effective concentration of vascular endothelial growth factor (VEGF) in angiogenesis is about 5000 pg/ml, while the detectable concentration in MSC-CM is only about 217 pg/ml [13]. A similar finding was observed in another study, which reported that CM derived from BM-MSCs overexpressed VEGF effectively repaired the hamster heart failure while CM from non-modified BM-MSCs is insufficient to improve the ventricular function, suggesting that conventional MSC-CM might not contain an adequate level of therapeutic paracrine factors [14]. Moreover, in our previous study, we also showed that inflammation pre-activation or gene modification enhanced the paracrine effects of BM-MSCs on intestinal injury, while non-activated MSC-CM was unable to exert therapeutic effect due to low concentration of the trophic factors [12, 15].

One strategy to solve the problem is to enhance the paracrine effect of MSCs. It has been demonstrated that a variety of stimuli and conditions can improve the paracrine effect of MSCs including pro-inflammatory stimuli, hypoxic preconditioning, gene modification [12, 16–19]. Ginsenoside RG1 is considered to be one of the most active ingredients in ginseng, possessing the ability of anti-inflammatory and immune-modulating effects [20–22]. It has been found to not only protect the MSCs from damage such as oxidative stress, but also enhance the proliferation and differentiation of MSCs [20, 22–24]. Moreover, recent study demonstrated that RG-1 enhanced the vascular endothelial growth factor (VEGF) secretion of adipose-derived stromal cells (ASCs), suggesting the RG-1 might be an attractive candidate to enhance the paracrine effect of MSCs [25].

Given the potential role of RG1 on the secretions of MSCs, we used RG1 to activate BM-MSCs and examined 1) the effect of RG-1 on paracrine potential of BM-MSCs. 2) the protective role of RG1-MSC-CM on RIII and the mechanism involved.

## RESULTS

### RG1 enhances BM-MSCs paracrine effects on irradiated IEC-6 cells in a dose-depend manner

**Figure 1:**
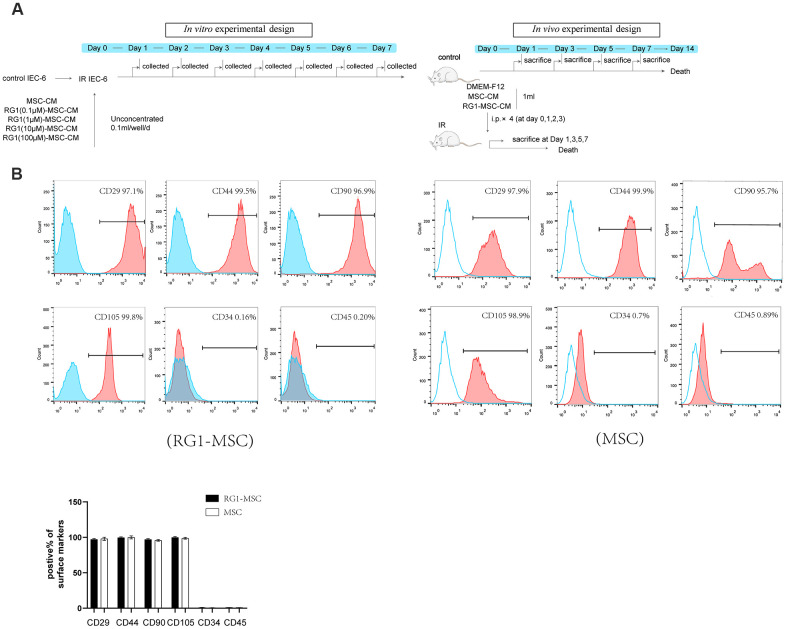
**RG-1 pre-activates bone marrow-derived mesenchymal stem cells (BM-MSCs) without altering the phenotype of BM-MSCs.** (**A**) *In vitro* and *in vivo* experiment design. *In vitro* design: IEC-6 irradiated with 10 Gy were treated with conditioned medium from non-activated BM-MSCs (MSC-CM) or BM-MSCs pre-activated by different concentration of RG1 (RG1-MSC-CM) (0.1 μM, 1 μM, 10 μM, 100 μM), followed by cell viability test every day until day 7. *In vivo* experiment: Sprague-Dawley rats were randomly divided into not irradiated (control) or irradiated group (IR) with a 14 Gy abdominal radiation on day 0, followed by a course of intra peritoneal injection (1 ml/day for 4 days) with control medium (DMEM-F12), MSC-CM, RG1-MSC-CM. Rats were either sacrificed at 1, 3, 5, 7 day for histological examination or were sacrificed at 1, 3, 7 day for functional evaluation. The remaining rats were used for survival analysis throughout a 14-day experiment. (**B**) Flow cytometry showed that BM-MSCs pre-activated by RG1 were positive for CD29, CD44, CD90, CD105 and negative for CD34, CD45. Data represent the mean ± SD (n=3) and were analyzed by t-test or one-way ANOVA.

*In vivo* and *in vitro* study designs are showed in Figure 1A. To evaluate the phenotype of BM-MSCs pre-activated by RG-1, we performed flow cytometry to identify the surface markers. Similar to the phenotype of BM-MSCs in our previous study [12], flow cytometry showed that CD29, CD44, CD90, CD105 were positive while CD34 and CD45 were negative in BM-MSCs activated by RG1 (Figure 1B), suggesting BM-MSCs still maintained its phenotype after activation of RG-1.

**Figure 2:**
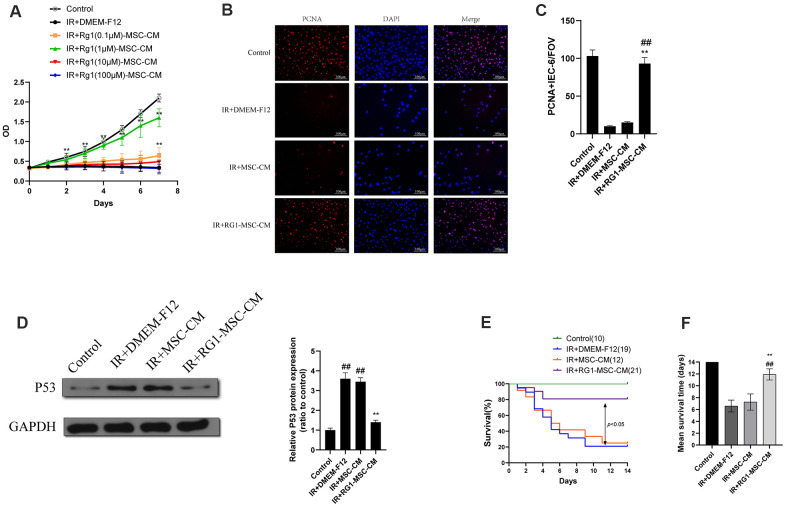
**RG1 enhances BM-MSCs paracrine effects on irradiated IEC-6 cells in a dose-depend manner. (A**)Cell proliferation of irradiated IEC-6 cell treated with DMEM-F12, MSC-CM or CM from BM-MSCs pre-activated by different concentration of RG1 (0.1 μM, 1 μM, 10 μM, 100 μM) is detected by CCK8 from day 0 to day 7. Data represent mean ± SD of three independent experiments. ^**^, *P* < 0.05 versus IR + DMEM-F12. (**B**) Immunofluorescence staining of IEC-6 cells with PCNA 3 days after radiation. Scale bars 100 μm. (**C**) Quantification of PCNA-positive cells. Data are reported as mean ± SD for 10 random fields per wells from at least three replicate wells per group. FOV, field of view. ^**^, *P* < 0.05 versus IR + DMEM-F12. ^##^, *P* < 0.05 versus IR + MSC-CM (**D**) The protein levels of p53 in IEC-6 were detected by western blot assays 3 days after radiation with GAPDH as the internal control. Data represent the mean ± SD (n=3). ^**^, *P* < 0.05 versus IR + DMEM-F12. ^##^, *P* < 0.05 versus Control. (**E**) Cumulative survival for rats exposed to 14 Gy abdominal irradiation by infusing with DMEM-F12, MSC-CM or RG1-MSC-CM (1 μM RG1) was analyzed using the Kaplan-Meier method. The cumulated number of rats in each experimental group is presented in parenthesis. *P*-values were determined by log-rank testing. (**F**) Mean survival time. Data represent the mean ± SD. ^**^, *P* < 0.05 versus IR + DMEM-F12. ^##^, *P* < 0.05 versus IR + MSC-CM. All data were analyzed by t-test or one-way ANOVA except as otherwise indicated.

To examine the effect of RG-1 on paracrine activity of BM-MSCs, we examined the effect of CM from BM-MSCs pre-activated by different concentration of RG-1 on irradiated IEC-6. IEC-6 cells in ionizing radiation (IR) +DMEM-F12 group and RG1(100μM)-MSC-CM group exhibited decreased cell viability, while RG1(0.1μM)-MSC-CM group and RG1(1μM)-MSC-CM group showed significant increase in cell proliferation, with the highest improvement in 1 μM RG1 group. Additionally, there was only a limited increase of optical density (OD) value in RG1(10μM)-MSC-CM group (Figure 2A). These findings suggested that 1 μM RG1 is the optimal dose to enhance the paracrine effect of BM-MSCs on RIII, which is used in further study.

To further evaluate the effect of RG1-MSC-CM on irradiated IEC-6, we performed PCNA immunofluorescence staining on irradiated IEC-6 and evaluated the expression of p53, which is a maker of DNA damage induced by radiation [26]. While IR+DMEM-F12 and MSC-CM group exhibited decreased expression of PCNA, RG1-MSC-CM significantly improved the expression of PCNA in irradiated IEC-6 (Figure 2B, 2C). Also, the expression of p53 was significantly higher in IR+DMEM-F12 group and IR+MSC-CM group, while RG1-MSC-CM attenuated the increase of p53 (Figure 2D), suggesting the RG1-MSC-CM improved the DNA damage caused by radiation.

### RG1-MSC-CM improves survival of rats exposed to 14-Gy abdominal irradiation via attenuating structural and functional damage of the intestine

After abdominal exposure to 14 Gy radiation, RG1-MSC-CM, MSC-CM and DMEM-F12 were infused into the abdomen of the experimental rats daily for 4 days respectively (Figure 1A). As seen in Figure 2E, 2F, RG1-MSC-CM significantly improved the survival of irradiated rats (*P* < 0.05) when compared to MSC-CM group and DMEM-F12 group. Though rats in the MSC-CM group have longer survival time than those in IR+DMEM-F12 group, the difference was not statistically significant (*P* > 0.05). These findings suggested the potential therapeutic effect of RG1-MSC-CM on the RIII.

**Figure 3:**
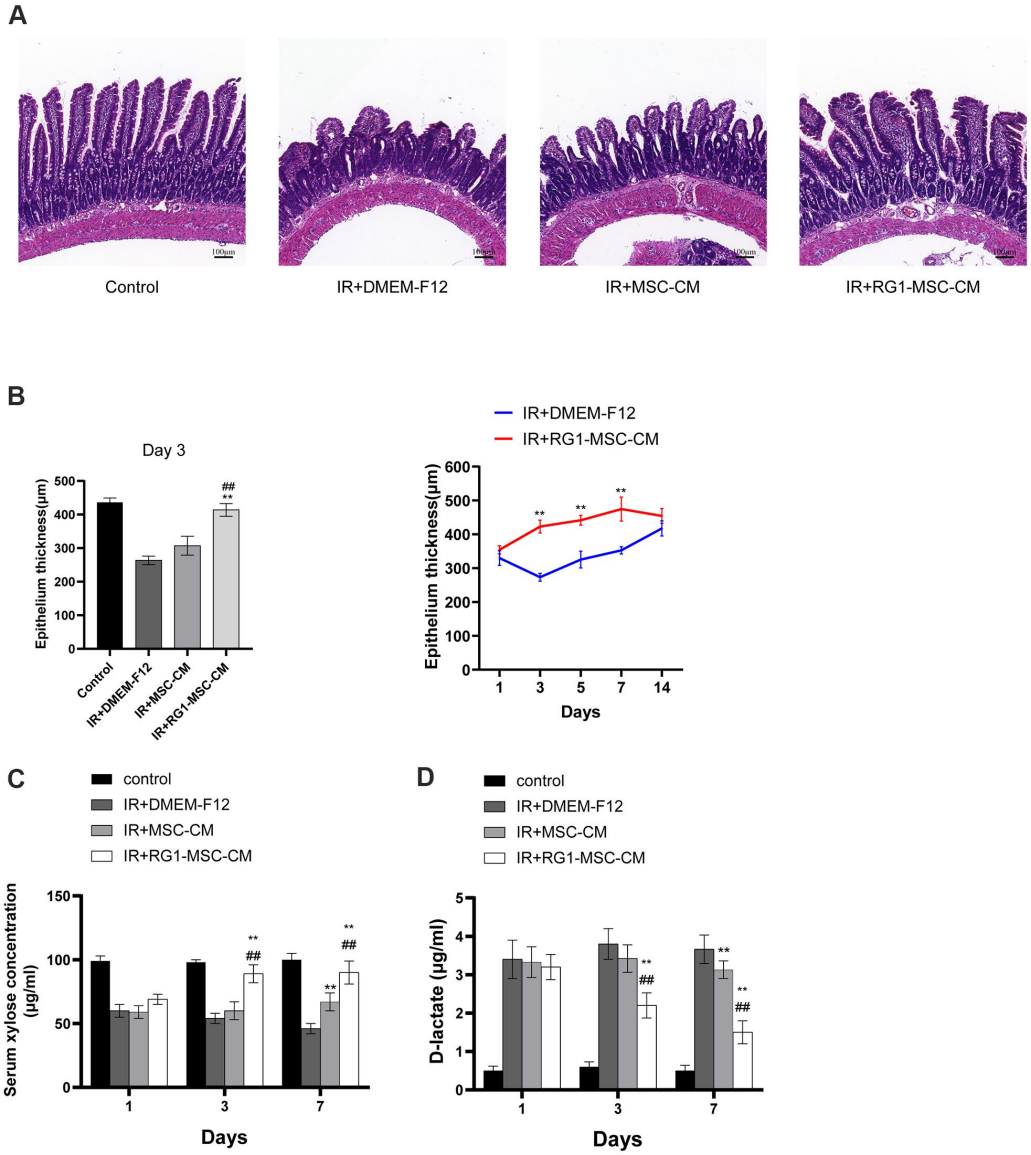
**RG1-MSC-CM improves intestinal function and structure of irradiated rats.** (**A**) Histological analysis by H&E staining. Scale bars 100 μm, magnification 10×. (**B**) Morphometric evaluation of epithelium thickness. Each value represents the average of 20 independent measurements per animal (5 animals per group). Value is reported as mean ± SD. ^**^, *P* < 0.05 versus IR + DMEM-F12, ^##^, *P* < 0.05 versus IR + MSC-CM. (**C**) Absorption function of intestines in irradiated rats was evaluated by D-xylose absorption experiment on 1, 3, 7 days after radiation. Data represent mean ± SD, ^**^, *P* < 0.05 versus IR + DMEM-F12, ^##^, *P* < 0.05 versus IR + MSC-CM. Data represent mean ± SD. (**D**) Intestinal permeability in rats was measured by plasma level of D-lactate on 1, 3, 7 days after radiation. Data represent mean ± SD. ^**^, *P* < 0.05 versus IR + DMEM-F12, ^##^, *P* < 0.05 versus IR + MSC-CM. All data were analyzed by t-test or one-way ANOVA except as otherwise indicated.

We further investigated the structural and functional change in rats. To examine the effect of CM on structural damage in RIII, intestinal segments were stained by Hematoxylin-eosin. Histological evaluation in post-irradiation period revealed necrotic epithelia, atrophy villi, inflammatory cell infiltration and a decrease in the number of crypts (Figure 3A). The pathohistological damage of the intestine was improved in the RG1-MSC-CM group, with increase in epithelium thickness compared to MSC-CM group and DMEM-F12 group (Figure 3A). With application of RG1-MSC-CM, the epithelium thickness was improved on day 3 and almost recovered to the same level as the control group on day 5, while those with MSC-CM only exhibited a slight change in the villi thickness (Figure 3B).

To evaluate the effect of CM on functional change in irradiated rats, serum xylose levels and plasma D-latate levels were used to evaluate the absorption function and permeability of the intestine respectively. As demonstrated in Figure 3C, 3D, irradiated rats treated with DMEM-F12 exhibited a great reduction in serum xylose levels and an increase in the plasma D-latate levels when compared to the control group, indicating the impairment of absorption function and intestinal permeability induced by radiation (Figure 3C, 3D). However, this effect was minimized by delivering RG1-MSC-CM illustrated by more functional recovery in RG1-MSC-CM group (*P* < 0.05). Such improvement in serum xylose levels and plasma D-latate levels were also observed in MSC-CM group in comparison to DMEM-F12 group, but it didn’t reach statistical significance until day 7 (Figure 3C, 3D). Taken together, RG1-MSC-CM remarkably improved structural abnormality as well as functional damage in RIII.

### RG1-MSC-CM attenuates apoptosis and promotes proliferation in the intestine

**Figure 4:**
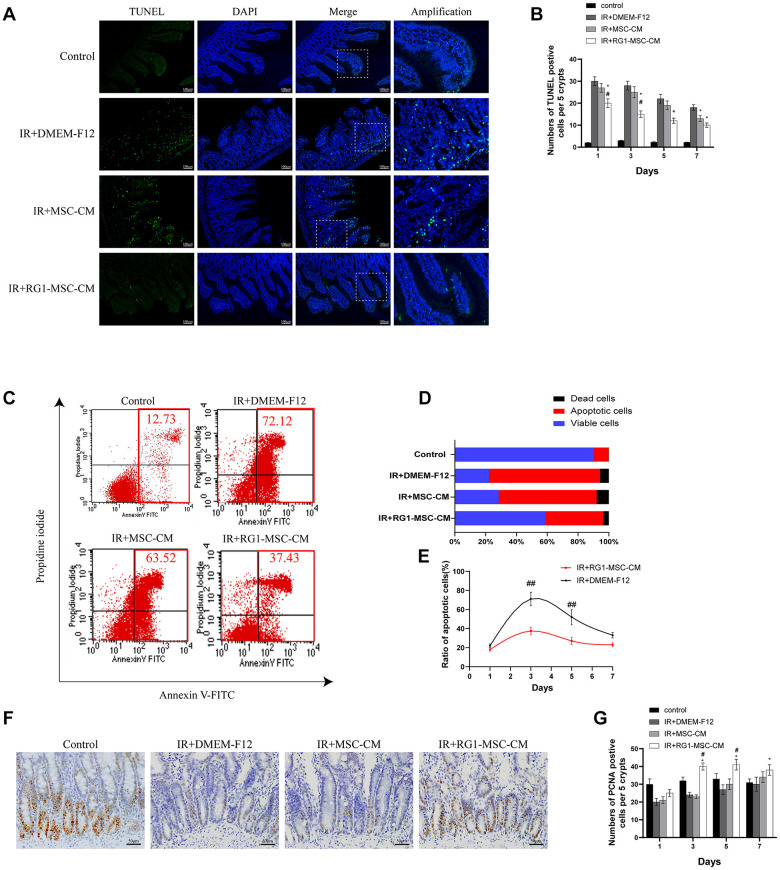
**RG1-MSC-CM attenuates apoptosis and promotes proliferation in the intestine.** (**A**) Apoptosis was assayed by TUNEL staining on day 3 of experiment. Scale bars 100 μm. (**B**) Quantification of TUNEL-positive cells on day 1, 3, 5, 7. n = 3 in each group. The number of positive cells in 5 crypts was scored in 100 crypts per section and reported as mean ± SD. ^*^, *P* < 0.05 versus IR + DMEM-F12. ^#^, *P* < 0.05 versus IR + MSC-CM. (**C**) Apoptosis of IEC-6 was evaluated by flow cytometry after PI/Annexin V staining on day 3 after radiation. The left upper quadrant contains necrotic cells (%); The upper right quadrant contains late apoptotic cells (%); The lower left quadrant contains live cells (%); and the lower right quadrant contains early apoptotic cells (%). (**D**) The percentage of total apoptotic cells, viable cells and dead cells under each condition are shown. (**E**) The ratio of apoptotic IEC-6 cells was determined by PI/Annexin V staining at 1, 3, 5, 7 days after radiation. Data represent mean ± SD of three independent experiments. ^##^, *P* < 0.05 versus IR+ DMEM-F12. (**F**) The proliferation of intestinal epithelial cells was examined by immunohistochemical staining with proliferating cell nuclear antigen (PCNA). Intestinal tissue samples were collected and analyzed on day 3 of experiment. Scale bars 50 μm. (**G**) Quantification of PCNA-positive cells on day 1, 3, 5, 7. n = 3~5 in each group. The number of positive cells in 5 crypts was scored in 100 crypts per section and reported as mean ± SD. ^*^, *P* < 0.05 versus IR + DMEM-F12. ^#^, *P* < 0.05 versus IR + MSC-CM. All data were analyzed by t-test or one-way ANOVA except as otherwise indicated.

The number of TUNEL-positive nuclei was used to indicate the severity of apoptosis. Compared to the control group, abdominal irradiation caused an approximately 10-fold increase of apoptotic cells on day 3 (Figure 4A). Treatment of RG1-MSC-CM significantly decreased the negative effect of RIII illustrated by reducing the number of TUNEL-positive nuclei on day 1, 3, 5 and 7 compared to IR+MSC-CM and IR+DMEM-F12 group (Figure 4B, *P* < 0.05). Despite the number of apoptotic cells was also reduced in IR+MSC-CM group, no significant differences were found in comparison to IR+DMEM-F12 group until day 7 (Figure 4B, *P* > 0.05).

To rule out the possibility that the inhibition of apoptosis by RG1-MSC-CM is mediated by indirect effect on immune system, we evaluated the effect of RG1-MSC-CM on RIII in *vitro*. Co-culturing with RG1-MSC-CM rather than MSC-CM significantly reduced the cellular apoptosis of irradiated IEC-6, as revealed by Annexin V/PI double-staining (Figure 4C, 4D). Moreover, RG1-MSC-CM significantly inhibited the rate of apoptotic cells in irradiated IEC-6 on day 3 and day 5 (*P* < 0.05), with no prominent effect on day 7 (Figure 4E).

To investigate the cellular proliferation, intestinal tissue samples were collected on day 1, 3, 5, 7 and examined after immunohistochemical staining with PCNA. With the treatment of RG1-MSC-CM, the number of PCNA-positive cells was significantly increased in comparison to those in IR+MSC-CM and IR+DMEM-F12 group on day 3, day 5 and day 7. However, the increase was not prominent in MSC-CM group (Figure 4F, 4G, *P* > 0.05).

### RG1-MSC-CM downregulates radiation-induced inflammatory responses *in vivo* and *in vitro*

**Figure 5:**
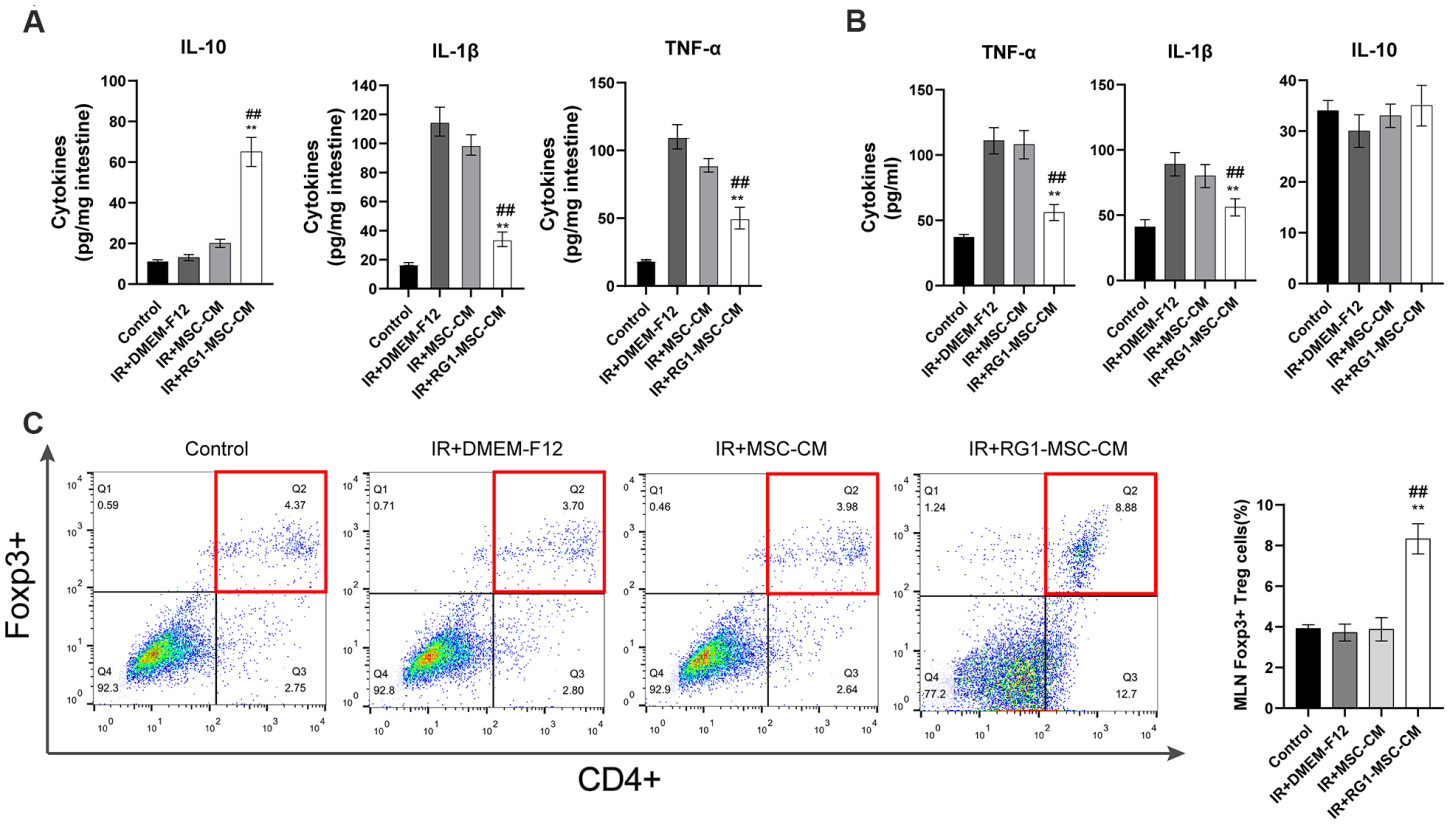
**RG1-MSC-CM downregulates inflammatory responses induced by radiation *in vivo* and *in vitro*.** (**A**) Pro/anti-inflammatory cytokines were extracted from jejunal protein of irradiated rats on day 3. The level of cytokines was determined by enzyme-linked immunosorbent assay (ELISA). (3~4 rats/group). ^**^, *P* < 0.05 versus IR + DMEM-F12. ^##^, *P* < 0.05 versus IR + MSC-CM. (**B**) Pro/anti-inflammatory cytokines of irradiated IEC-6 was measured by ELISA on day 3 after radiation. Data represent mean ± SD (n=3). ^##^, *P* < 0.05 versus IR + MSC-CM. ^**^, *P* < 0.05 versus IR + DMEM-F12. (**C**) The percentages of CD4^+^Foxp3^+^ Treg cells in the CD4^+^ population of mesenteric lymph nodes (MLNs) were determined by flow cytometry (4~5 rats/group). Tissue samples were collected on day 3 of experiment. Data represent the mean ± SD. ^##^, *P* < 0.05 versus IR + DMEM-F12. All data were analyzed by t-test or one-way ANOVA except as otherwise indicated.

To investigate the effect of RG1-MSC-CM on inflammatory responses on RIII, we evaluated IL-1β, TNF-α, IL-10 *in vivo* and *in vitro*. While radiation upregulated the level of pro-inflammatory factors (IL-1β and TNF-α), RG1-MSC-CM rather than MSC-CM significantly reversed the increase *in vivo* and *in vitro* (Figure 5A, 5B). Although the level of IL-10 in the mucosa was significantly increased by RG1-MSC-CM, the effect was not prominent *in vitro* (Figure 5B). To further investigate the immunomodulatory of RG1-MSC-CM, we collected tissue samples from the mesenteric lymph nodes (MLNs) on day 3. As shown in Figure 5, the percentages of CD4^+^Foxp3^+^ Treg cells in the MLNs were significantly increased with the treatment of RG1-MSC-CM, suggesting RG1-MSC-CM induced a downregulation of local proinflammatory response after irradiation (Figure 5C).

### RG1-MSC-CM promotes angiogenesis of intestine in the irradiated rats

**Figure 6:**
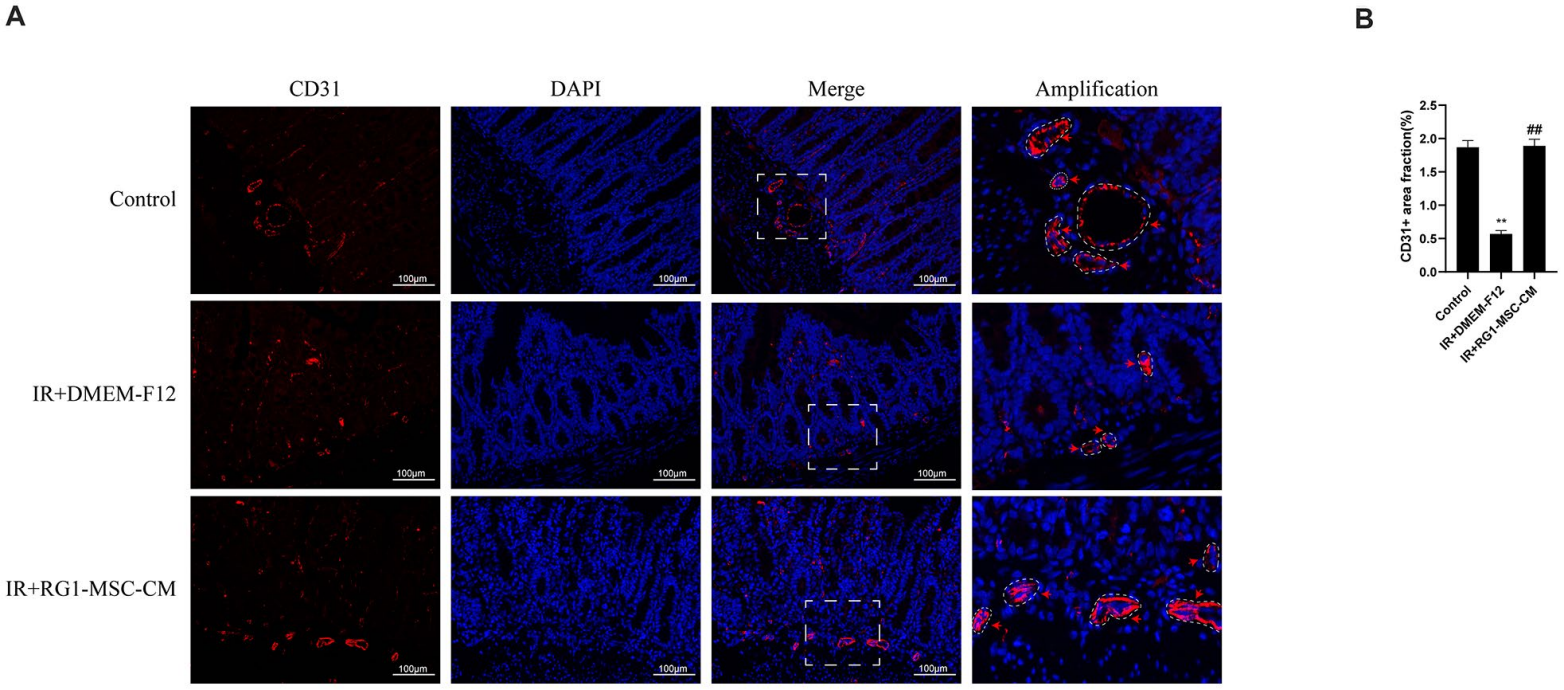
**RG1-MSC-CM improves the angiogenesis of intestine in irradiated rats.** (**A**) Immunofluorescence staining with CD31. Intestinal samples were collected and stained on day 3 after radiation. Arrowheads indicate a CD31-positive area. Scale bars 100 μm. (**B**) Quantification of CD31^+^ area fraction. Data represent mean ± SD for “hot spot” area. Each group contains at least 3 rats (3-4 section/rat). ^##^, *P* < 0.05 versus IR + DMEM-F12. ^**^, *P* < 0.05 versus Control. All data were analyzed by t-test or one-way ANOVA except as otherwise indicated.

To investigate the effect of RG1-MSC-CM on the angiogenesis of intestine of irradiated rats, immunofluorescence staining for CD31, a pan-endothelial marker representing the extent of angiogenesis [27], was performed on day 3 after radiation. As shown in Figure 6, the CD31-positive area fraction in IR+DMEM-F12 group was significantly decreased compared to that in control group, suggesting the impaired angiogenesis induced by radiation. With administration of RG1-MSC-CM, the area fraction of CD31 was greatly increased compared to that in IR+DMEM-F12 group (*P* < 0.05). No statistical difference was observed between IR+MSC-CM group and IR+DMEM-F12 group. (Figure 6A, 6B). Taken together, these findings suggested RG1-MSC-CM promotes the angiogenesis of intestine in the irradiated rats.

### RG1-MSC-CM improves regeneration of intestinal stem cells (ISCs) in the irradiated rats

**Figure 7:**
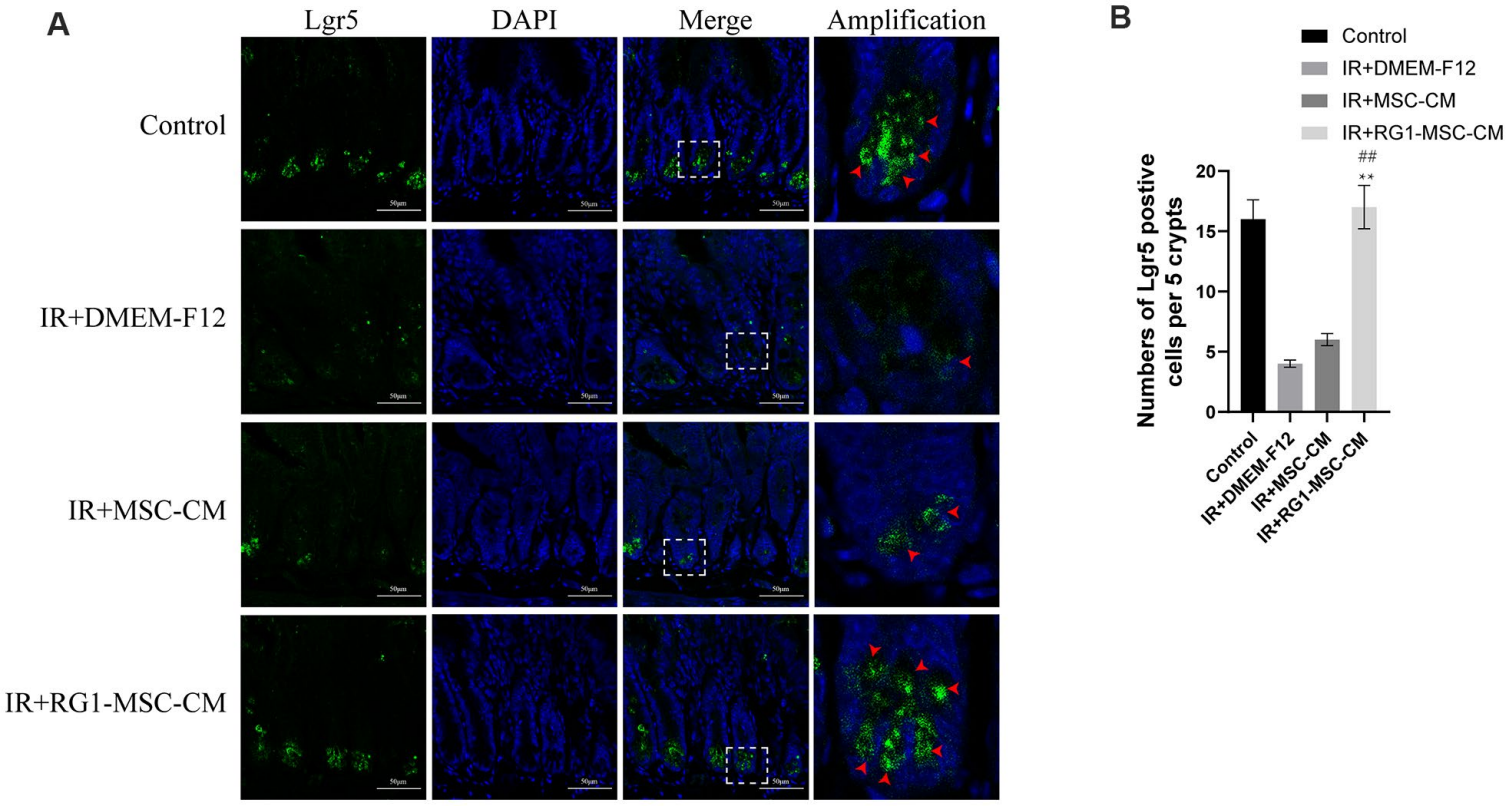
**RG1-MSC-CM improves regeneration of intestinal stem cells (ISCs) in the irradiated rats.** (**A**) Immunofluorescence staining with Lgr5. Intestinal samples were collected and stained on day 3 after radiation. Arrowheads indicate Lgr5-positive cells. Scale bars 50 μm. (**B**) Quantification of Lgr5-positive cells. n = 3~5 in each group. The number of positive cells in 5 crypts was scored in 100 crypts per section and reported as mean ± SD. ^**^, *P* < 0.05 versus IR + DMEM-F12. ^##^, *P* < 0.05 versus IR + MSC-CM. All data were analyzed by t-test or one-way ANOVA except as otherwise indicated.

Intestinal cell marked by Leucine-rich repeat-containing G protein-coupled receptor 5 (Lgr5) is a kind of ISCs responsible for modulating epithelium homeostasis, renewal and regeneration after injury [28]. Immunofluorescence staining showed that 3 days radiation significantly decreased the number of Lgr5 positive cells in the crypts compared to control group, with 0~2 Lgr5-positive cells localized to the crypts. While MSC-CM caused limited improvement in the expression of Lgr5, RG1-MSC-CM significantly improved the number of Lgr5 positive cells. Taken together, these findings suggested that RG1-MSC-CM increased the proliferation of ISCs in the intestine after radiation. (Figure 7).

### RG1-MSC-CM promotes RIII recovery via increased release of VEGF and IL-6

**Figure 8:**
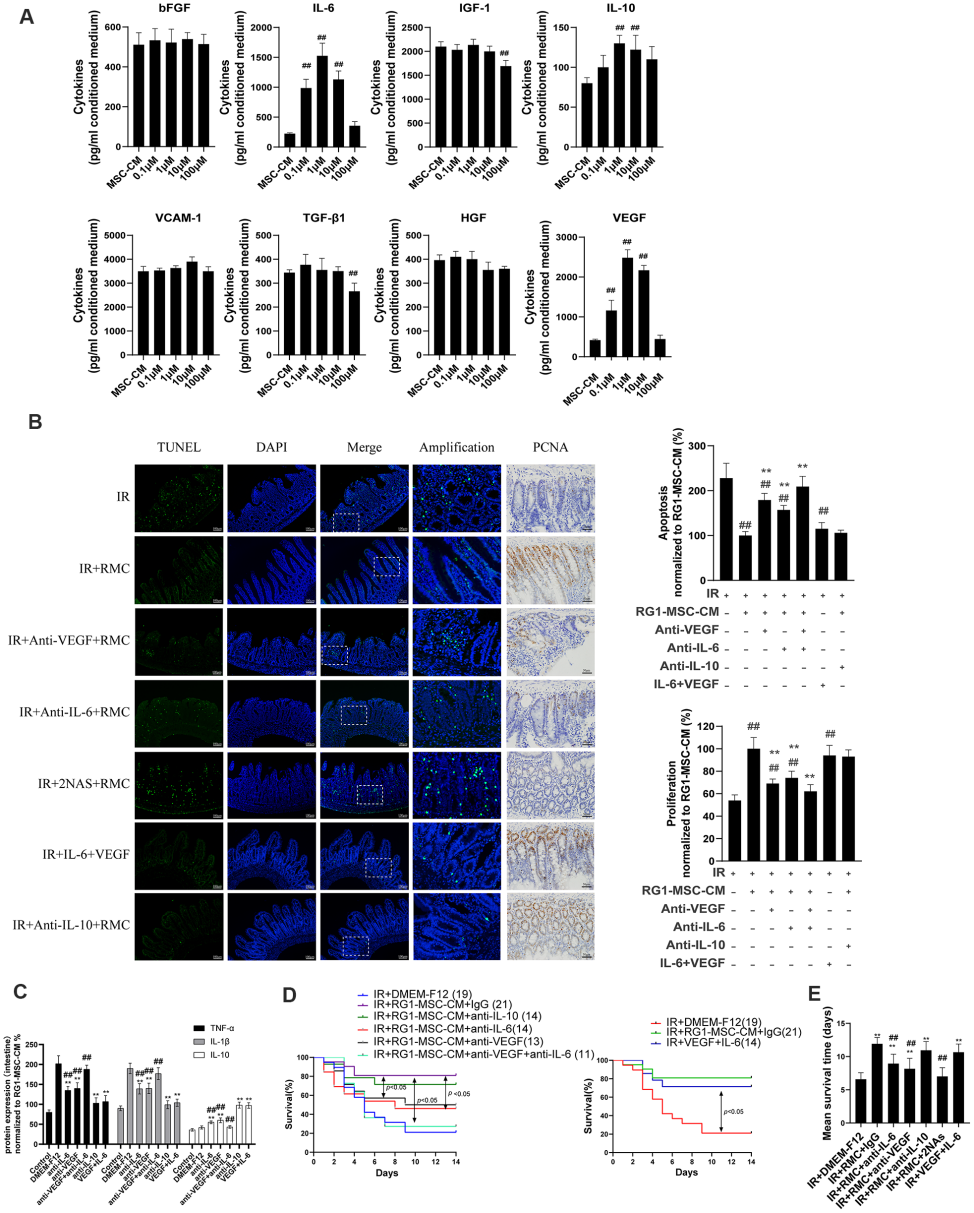
**RG1-MSC-CM promotes RIII recovery via increased release of VEGF and IL-6.** (**A**) 8 selected cytokines in unconcentrated conditioned medium derived from BM-MSCs pre-activated by different concentration of RG1 (0.1 μM, 1 μM, 10 μM, 100 μM) were detected by ELISA. Data represent mean ± SD of at least three independent experiments. ^##^, *P* < 0.05 versus MSC-CM. (**B**) The effect of neutralization of 3 selected cytokines or addition of selected exogenous cytokines on apoptosis (left panel) and proliferation (right panel) of intestine in irradiated rats. The apoptosis and proliferation were evaluated by quantification of TUNEL-positive and PCNA-positive cell per 5 crypts 3 days after radiation, respectively. Data is reported as mean ± SD (n = 4~5). ^##^, *P* < 0.05 versus IR + DMEM-F12. ^**^, *P* < 0.05 versus IR + RG1-MSC-CM. RMC: RG1-MSC-CM, 2NAs: anti-VEGF antibody + anti-IL-6 antibody. Scar bar 50 μm and 100 μm. (**C**) Pro/anti-inflammatory cytokines were extracted from jejunal protein of irradiated rats on day 3. The level of cytokines was determined by enzyme-linked immunosorbent assay (ELISA). (3~4 rats/group). ^**^, *P* < 0.05 versus IR + DMEM-F12. ^##^, *P* < 0.05 versus IR + RG1-MSC-CM. (**D**) The effect of neutralization (left panel) of selected cytokines or addition of selected exogenous (right panel) cytokines on the survival of irradiated rats. Cumulative survival analyzed using the Kaplan-Meier method. P-values were determined by log-rank testing. The number of rats in each group is present in the parenthesis. (**E**) Mean survival time. Data represent the mean ± SD. ^**^, *P* < 0.05 versus IR + DMEM-F12. ^##^, *P* < 0.05 versus IR + RG1-MSC-CM.

To explore the mechanism in RG1-mediated paracrine activity, we further analyzed the alternation of secreting factors in CM from BM-MSCs or RG1-MSCs. Based on our previous cytokines array of conditioned medium derived from BM-MSCs [12], we analyzed 8 secreting factors highly expressed in MSC-CM (bFGF, IL-6, IGF-1, IL-10, VCAM-1, TGF-β1, HGF, VEGF) by ELISA which have been reported to improve tissue regeneration in RIII [1, 29]. Consistent with the effect on cell viability of IEC-6, the dose of RG1 lower than 1 μM (0.1 μM, 1 μM) enhanced 3 selected cytokines secretion of BM-MSCs (VEGF, IL-6, IL-10) with the optimal dose of 1 μM (Figure 8A). In RG1-MSC-CM (1μM) group, the concentration of VEGF, IL-6, IL-10 was 2484.2 ± 200.6 pg/ml, 1525.6 ± 212.5 pg/ml and 130.0 ± 10.7 pg/ml respectively, which were approximately 6, 5, 1.5 times higher than those in MSC-CM respectively (Figure 8A).

Although the ELISA assay confirmed the presence of 5 selected cytokines (bFGF, VCAM-1, HGF, TGF-β1, IGF-1) in RG1-MSC-CM, their concentrations were not significantly higher than those in MSC-CM (Figure 8A). Notably, 2 cytokines (IGF-1, TGF-β1) in 100 μM RG-1 group were significantly lower than MSC-CM group (Figure 8A). These findings suggested that RG1 might pre-activate the paracrine potential of BM-MSCs in a dose-dependent manner, while dose higher than 100 μM might hamper the paracrine activity of BM-MSCs (Figure 8A).

Given that 3 cytokines (VEGF, IL-6, IL-10) were elevated in RG1-MSC-CM compared to MSC-CM, we further examined whether the RG1-MSC-CM exerted its therapeutic effect through these 3 cytokines. As showed in Figure 8, the therapeutic effect of RG1-MSC-CM on apoptosis, proliferation, inflammation response and survival of irradiated rats was partially inhibited by antibodies against VEGF or IL-6 individually and further blocked by combined neutralization of VEGF and IL-6, while the neutralization against IL-10 didn’t influence the protective effect of RG1-MSC-CM (Figure 8B–8E). In addition, combined injection of IL-6 and VEGF could exhibit a similar therapeutic effect as treated with RG1-MSC-CM (Figure 8B–8E). These indicated that a pivotal role of IL-6 and VEGF in RG1-MSC-CM mediated RIII recovery.

### The therapeutic effect of IL-6 and VEGF is partially mediated via HO-1 dependent mechanism

**Figure 9:**
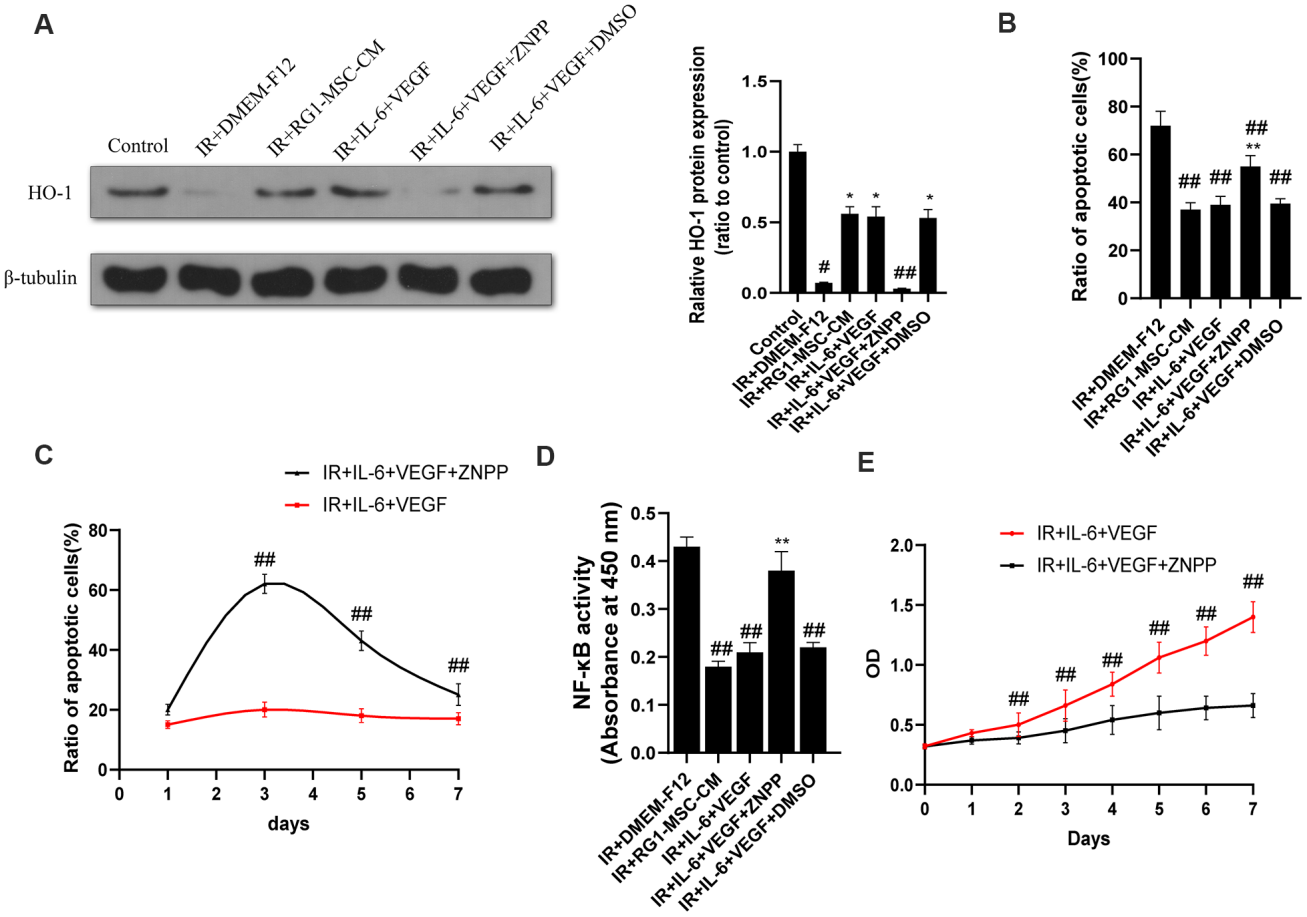
**The therapeutic effect of IL-6 and VEGF is partially mediated via HO-1 dependent mechanism.** (**A**) The protein levels of HO-1 in IEC-6 were detected by western blot assays on day 3 after radiation with β-tubulin as the internal control. Data represent the mean ± SD (n=3). ^*^, *P* < 0.05 versus IR + DMEM-F12. ^#^, *P* < 0.05 versus Control. ^##^, *P* < 0.05 versus IR + IL-6 + VEGF. (**B**) Inhibition of HO-1 partially inhibits the therapeutic effect of IL-6 + VEGF on apoptosis of IEC-6. The ratio of apoptosis IEC-6 cells was determined by PI/Annexin V staining on day 3 after radiation. Data represent mean ± SD of three independent experiments. ^**^, *P* < 0.05 versus IR + IL-6 + VEGF. ^##^, *P* < 0.05 versus IR + DMEM-F12. (**C**) The ratio of apoptosis IEC-6 cells was determined by PI/Annexin V staining on 1, 3, 5, 7 days of the experiment. Data represent mean ± SD of three independent experiments. ^##^, *P* < 0.05 versus IR+ IL-6 + VEGF. (**D**) The activity of NF-κB p65 in IEC-6 was measured by an ELISA-based assay directed against the p65 subunit of NF-κB. Data represent the mean ± SD (n=3). ^##^, *P* < 0.05 versus IR + DMEM-F12. ^**^, *P* < 0.05 versus IR + IL-6 + VEGF. (**E**) Cell viability was detected by CCK-8 from day 0 to day 7 after radiation. ^##^, *P* < 0.05 versus IR + IL-6 + VEGF + ZNPP. Data represent mean ± SD of three independent experiments. All data were analyzed by t-test or one-way ANOVA except as otherwise indicated.

As induction of HO-1 has been reported to ameliorate RIII and IL-6/VEGF can modulate the expression of HO-1 [30–33], We next investigated the effect of IL-6 and VEGF on HO-1 expression *in vitro*. As shown by western blot, radiation significantly downregulated the expression of HO-1 in IEC-6 compared to control group (*P* < 0.05) (Figure 9A). The expression of HO-1 was significantly upregulated in RG1-MSC-CM and IL-6+VEGF group compared to IR+DMEM-F12 group (*P* < 0.05) (Figure 9A), and this was reversed by ZNPP. To further investigate whether HO-1 is responsible for the therapeutic benefit of IL-6+VEGF in RIII, we evaluated apoptosis, NF-kB binding activity, a maker of acute gut mucosa inflammation [34], and cell viability of IEC-6. As shown in Figure 9B–9E, the therapeutic effects of IL-6+VEGF on apoptosis, NF-kB binding activity and the cell viability of IEC-6 were partially inhibited by ZNPP. These finding suggests that the therapeutic effect of IL-6 and VEGF on RIII is at least partially mediated in a HO-1 dependent mechanism. (Figure 9B–9E).

### The superior paracrine effect of RG1-MSC is partially mediated by HO-1

**Figure 10:**
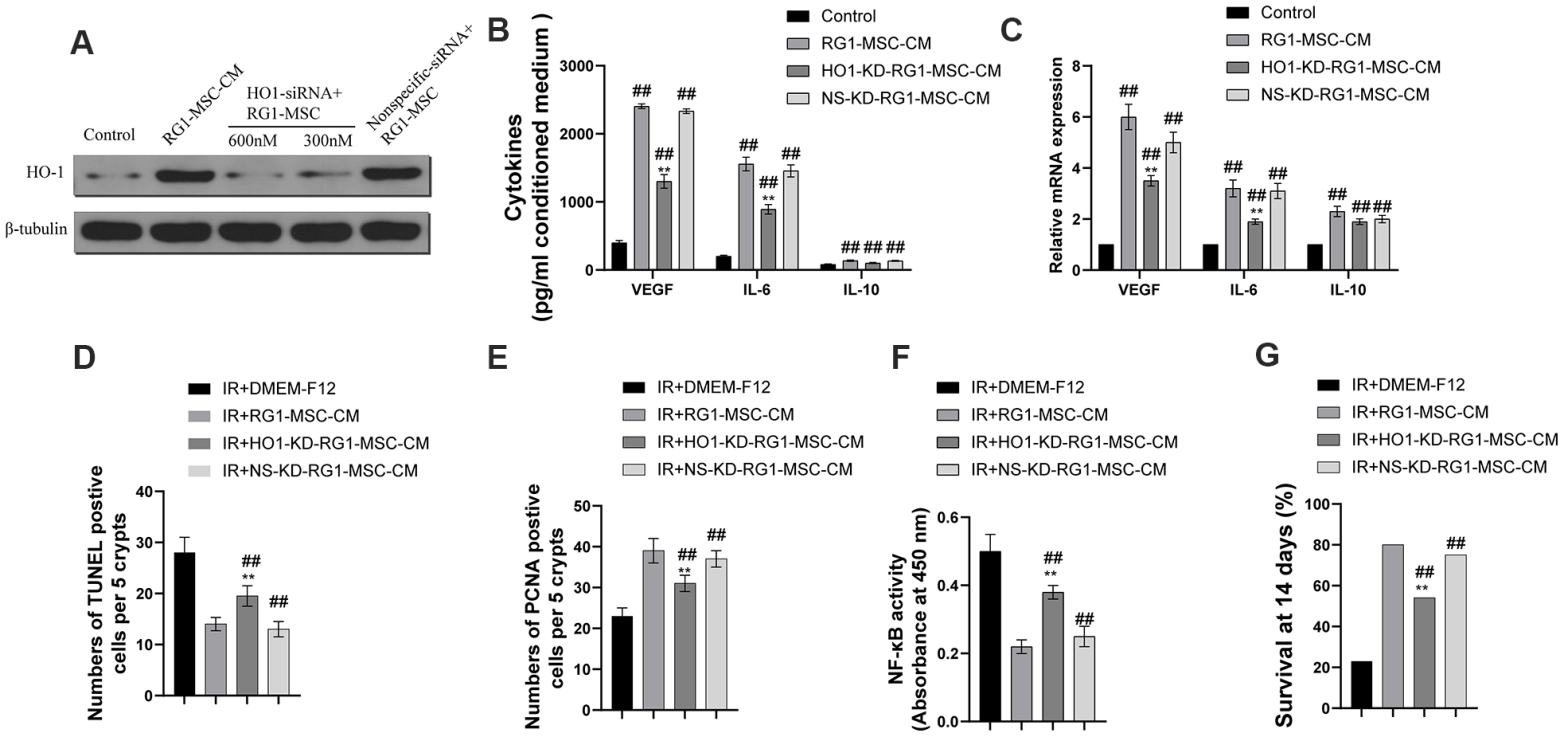
**Heme oxygenase-1 mediates the difference in the secretome and the therapeutic effects of RG1-MSC-CM.** MSCs were transfected with varying doses of HO-1 siRNA and nonspecific siRNA prior to RG1 stimulation. The protein levels of HO-1 (**A**), the secretion (**B**) and the mRNA expression (**C**) of VEGF, IL-6 and IL-10 from BM-MSCs were evaluated. ^##^, *P* < 0.05 versus control, ^**^, *P* < 0.05 versus RG1-MSC-CM. NS-KD-RG1-MSC-CM: conditioned medium from non-specific siRNA transfected MSCs activated by RG1. HO-1-KD-RG1-MSC-CM: conditioned medium from HO-1 siRNA transfected MSCs activated by RG1. Administration of HO1-KD-RG1-MSC-CM partially abolished the therapeutic effect of RG1-MSC-CM on the apoptosis (**D**) and proliferation (**E**) of irradiated intestinal epithelial cells *in vivo*, which were evaluated by quantification of TUNEL-positive and PCNA-positive cells in the intestine, respectively. ^##^, *P* < 0.05 versus IR + DMEM-F12. ^**^, *P* < 0.05 versus IR + RG1-MSC-CM. (**F**) The activity of NF-κB p65 in intestine on day 3 of experiment was measured by an ELISA-based assay directed against the p65 subunit of NF-κB. Data represent the mean ± SD (n=3). ^##^, *P* < 0.05 versus IR + DMEM-F12. ^**^, *P* < 0.05 versus IR + RG1-MSC-CM. (**G**) The ability to improve the 14-days survival rate were partially reversed by administration of HO1-KD-RG1-MSC-CM. ^##^, *P* < 0.05 versus IR + DMEM-F12. ^**^, *P* < 0.05 versus IR + RG1-MSC-CM. All data were analyzed by t-test or one-way ANOVA except as otherwise indicated.

**Figure 11:**
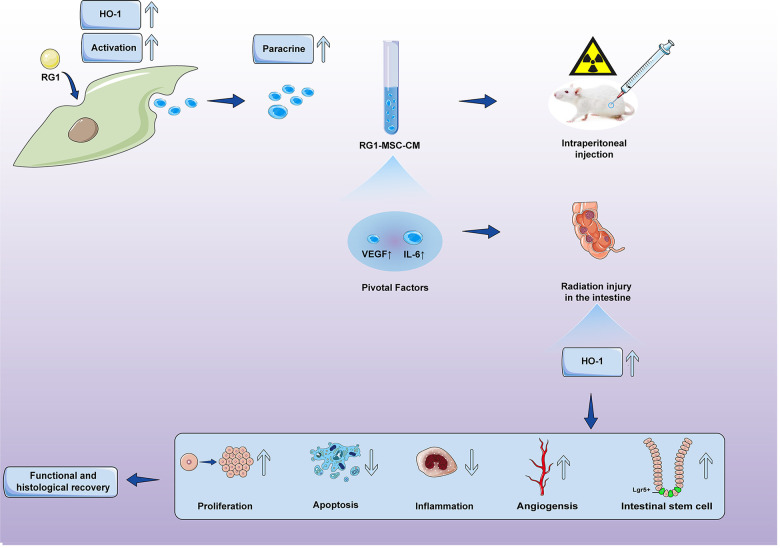
**Conditioned medium from BM-MSCs pre-activated by RG1 (RG1-MSC-CM) releases higher concentrations of VEGF and IL-6, and protects against RIII via improvement of intestinal regeneration, inflammation, angiogenesis and stem cell regeneration in a heme oxygenase-1 dependent mechanism.**

As previous studies have demonstrated that over expression of HO-1 enhanced the paracrine effect, anti-apoptotic and anti-inflammatory ability of BM-MSCs, we further explored whether HO-1 plays an important part in RG1-mediated superior paracrine effect on RIII. As shown in Figure 10A, RG1 significantly increased the expression of HO-1, while 600 nmol/L HO-1 siRNA remarkably downregulated HO-1 expression, with no decrease in NS-KD-RG1-MSC-CM group (Figure 10A). To further explore the role of HO-1 in the superior paracrine effect of RG1-MSC, we detected the secretion and mRNA of 3 upregulated paracrine factors from BM-MSCs. Knockdown of HO-1 in BM-MSCs partially abolished the expression of VEGF, IL-6 in both secretion and mRNA expression (Figure 10B, 10C). The discovery *in vitro* promoted us to further explore whether the change could be implemented *in vivo*. While RG1-MSC-CM improved the intestinal proliferation, apoptosis and NF-kB binding activity as well as survival of irradiated rats, knockdown of HO-1 in BM-MSCs partially abolished these effect (Figure 10D–10G). These results suggested that the superior paracrine effect of RG1-MSC in RIII is partially mediated by HO-1 (Figure 11).

## DISCUSSION

Our study confirmed the hypothesis that RG1 can enhance the paracrine effects of BM-MSCs on RIII and showed that 1) RG1-MSC-CM, but not MSC-CM, improved survival and promoted the structural and functional restoration of RIII by regulating intestinal regeneration, inflammation and angiogenesis. 2) RG1 enhanced the BM-MSCs secretion of VEGF and IL-6 therefore providing superior therapeutic efficacy on RIII. 3) the protective effect of IL-6 and VEGF on RIII is partially mediated via HO-1 dependent mechanism. 4) the superior paracrine effect of RG1-MSC is partially mediated by upregulation of HO-1.

There are some important observations of our work. First, we demonstrated that RG1 enhanced the paracrine potential of BM-MSCs. Ginsenoside RG1 has been showed to optimize the biological feature of MSCs. Previous studies have demonstrated that RG-1 plays an importance role in protecting stem cell from damage such as radiation injury and promoting the proliferation and differentiation of MSCs [35–38]. Recently, a study reported that RG-1 can enhance the paracrine activity of ASCs *in vitro* [25]. In our study, we revealed that RG-1 enhanced the paracrine activity of BM-MSCs in a dose-dependent manner. 1 μM is the optimal dose for RG-1 to enhance paracrine activity of BM-MSCs. Opposite to lower concentration of RG-1, application of over-dosage RG-1 (≥100 μM) might inhibit the paracrine effect of BM-MSCs, consistent with previous findings that 100 μM RG-1 inhibits the biological function of ASCs and induces the apoptosis of mouse blastocyst cells [25, 39]. In conclusion, our study suggested that the optimum dose of RG1 for enhancing the paracrine activity of BM-MSCs is 1 μM and the safety threshold should be lower than 100 μM.

Second, we found that RG1-MSC-CM had a beneficial effect on RIII. As our study showed, RG1-MSC-CM, but not MSC-CM, provided protective effects on RIII via modulation of intestinal proliferation/apoptosis, inflammation, angiogenesis and stem cell regeneration. These findings are similar to those of MSCs transplantation in other studies [40–43], indicating that RG1-MSC-CM is a cell-free alternative as effective as MSCs transplantation.

In order to explore the mechanism underlying enhanced paracrine potential of RG1-MSCs, we detected whether there were differences of the paracrine factors in MSC-CM with and without RG1. We found that the level of VEGF and IL-6 were significantly higher in. RG1-MSC-CM Further neutralizing experiment confirmed that VEGF and IL-6 were pivotal factors in RG1-MSC-CM-mediated recovery in RIII. Combined infusion of VEGF and IL-6 has comparable therapeutic efficacy to RG1-MSC-CM, suggesting that the combined use of IL-6 and VEGF could be another promising therapeutic strategy on RIII. Our finding is consistent with previous studies that VEGF and IL-6 might play important roles in RIII. For example, IL-6 improves cellular proliferation and regulates crypt homeostasis in the intestine of murine colitis [44, 45], while blockage of IL-6 exacerbates acute and late injury of the intestine after focal radiation [46]. VEGF, a primary mediator of angiogenesis, restores the intestine after total-body irradiation [47] whereas administration of anti-VEGF antibody aggravates intestinal injury compared to irradiated mice [48].

Thirdly, we found that the superior paracrine effect of RG1-MSC on RIII is partially mediated by HO-1. HO-1 is a cytoprotective protein expressed at a very low level in the MSCs and can be induced by stimulation such as inflammation, drug [12, 49, 50]. It has been reported that overexpression of HO-1 enhanced the anti-apoptotic, anti-oxidative, immunomodulatory, paracrine ability of MSCs, while silence of HO-1 in MSCs reversed these effects [51–53]. We revealed that RG1 stimulation significantly upregulated the expression of HO-1 in BM-MSCs and the therapeutic effects were partially abolished with the use of HO-1 siRNA. Thus, the superior paracrine effect of RG1-MSC on RIII is partially mediated by HO-1. However, even after silencing HO-1 in BM-MSCs, the improved therapeutic efficacy as well as paracrine activity can still be observed, suggesting that additional mechanism need to be explored.

Finally, we explored the specific mechanism involved in the therapeutic effect of IL-6 and VEGF on RIII. Multiple studies have shown that HO-1 induction plays an important role in cellular protection against intestinal injury [30, 31, 54]. Moreover, previous studies have also shown that IL-6 or VEGF can stimulate the expression of HO-1 [32, 33, 55–57]. In our study, we observed that HO-1 were upregulated in both RG1-MSC-CM and VEGF+IL-6 group. Further blockage of HO-1 by ZNPP partially inhibited the therapeutic effect of IL-6 and VEGF, suggesting that the IL-6 and VEGF mediated-recovery is partially via HO-1 dependent mechanism.

Some limitations must be acknowledged in our studies. First, the different therapeutic effects between RG1-MSC-CM and BM-MSCs pre-activated by RG1 was not studied because previous study has demonstrated CM derived from pre-activated MSCs has therapeutic effect comparable to transplantation of MSCs pre-activated in treating intestinal injury [58, 59]. Second, conditioned medium from MSCs contains soluble factors and extracellular vesicles (EVs). This study only monitored the changes in MSC paracrine factors and their effects on RIII. Previous studies have showed the ability of EVs to improve intestinal injury [60, 61], suggesting that EVs may also play an important role in the CM-mediated recovery in RIII.

In conclusion, the present study showed that 1) RG1 enhances the paracrine efficacy of BM-MSCs through upregulation of HO-1. 2) RG1 increased the secretions of pivotal factors VEGF/IL-6 in BM-MSCs to promote recovery of RIII via HO-1 dependent mechanism. Our study revealed a new role of RG-1 on enhancing the biological function of BM-MSCs, offering an option in the stem cell therapy on RIII and even other tissue injury.

## MATERIALS AND METHODS

### Ethical approval

The methods were carried out in “accordance” with the approved guidelines.

### Animals

Adult Sprague-Dawley rats (8 weeks) were sourced from the Laboratory Animal Center of Sun Yat-Sen University (China), weighing between 280~350 g. For primary culture of BM-MSCs, Sprague-Dawley rats weighing between 60~100 g, 3~4 weeks, females were used. The whole procedure was conducted following guideline of our local animal care and use committee.

### Cell line and cell culture

BM-MSCs were collected from the femurs of the adult Sprague-Dawley rats. The obtained BM-MSCs were cultured in Dulbecco's Modified Eagle Media: Nutrient Mixture F-12 (DMEM-F12) containing 10% heat inactivated fetal bovine serum (FBS), 1% Glutamine, and 1% Penicillin/Streptomycin. The phenotype of BM-MSCs was characterized by flow cytometry at passage 3 and BM-MSCs were used for a maximum of 5 passages.

Rat intestinal epithelial IEC6 cells were obtained from the American Type Culture Collection (ATCC Cat# CRL-1592, RRID:CVCL_0343). Cells were cultured in DMEM, 5% FBS, 0.1 units/ml insulin and 1% Penicillin/Streptomycin. Cells before 20 passages were used. All cells were maintained in a humidified atmosphere of 5% CO2 at 37° C

### Radiation-induced intestinal injury (RIII) model

The experimental rats were anesthetized before irradiation. The radiation procedure was performed by exposing their abdomens to a linear accelerator (Siemens PRIMUS) at a dose rate of 300 cGy/min. Lead chamber was used to protect other parts of body from radiation [62]. Based on our previous experiment, 14 Gy was selected as the optimal irradiation dose for detecting the therapeutic action of MSC-CM [12]. Some of the rats were monitored for survival status throughout the two-week-course of the experiment while the remaining were used for tissue collection. Tissues samples were collected from the intestines of rats on day 1, 3, 5, 7 for structural and functional examination.

For *in vitro* study, IEC-6 cells irradiated with 10 Gy were collected every day until day 7 to evaluate cell viability.

For heme oxygenase-1 (HO-1) inhibition, IEC-6 was incubated with Zn-protoporphyrin (Zn-PP) 0.1 μM (Sigma, St. Louis, MO) [49].

### Preparation and treatment of conditioned medium

To prepare conditioned medium, BM-MSCs were pre-activated in cultured medium containing different concentration of RG1 (0.1 μM, 1 μM, 10 μM, 100 μM RG1, Sigma, St. Louis, MO, USA) for 7 days, which will be refreshed daily. Cells were passaged every 2~3 days. After 7 days culture, cultured medium was removed and BM-MSCs were conditioned in serum-free DMEM-F12 medium for 48h. The conditioned medium was then collected for ELISA assay to detect the concentration of cytokines. For further use, conditioned medium was concentrated 50-fold by ultrafiltration with a 5 kDa cut-off (Millipore, Billerica, MA) and stored at -20° C

To determine the optimized concentration of RG-1 on activation of BM-MSCs, IEC-6 cells (1×10^4^ cells/well) irradiated at 10 Gy were cultured in CM (0.1 ml/day/well) derived from BM-MSCs pre-activated by different concentration of RG-1 (0.1 μM, 1 μM, 10 μM, 100 μM) in 96-well plates. Cell viability were determined by CCK8 every day until day 7.

For *in vivo* study, rats were intraperitoneally injected with conditioned medium for 4 days (day 0, 1, 2, 3) (1 ml/day/rat).

For the neutralizing experiments, the conditioned medium was collected and incubated for 30 minutes with control IgG or individual neutralizing antibodies for VEGF (1 μg/ml; (R&D Systems Cat# AF564, RRID:AB_2212821)), IL-6 (1 μg/ml; (R&D Systems Cat# AF506, RRID:AB_355398)), IL-10 (1 μg/ml; (R&D Systems Cat# AF519, RRID:AB_355408)), or their combinations.

### Cell counting Kit-8 (CCK-8 test)

Cell proliferation was assessed followed the instruction (Dojindo Cell Counting Kit-8). IEC-6 cells were cultured in 96-well culture with density of 1×10^4^ cells/well for 24h. After 10Gy radiation and treatment, 10 ul of CCK-8 was added to each well and incubated at 37° C for 1 hours. Cell proliferation was measured as Optical Density (OD) at 450 nm using Multiskan Spectrum (Thermo Fisher).

### D-xylose concentrations test

Intestinal absorption function was measured by D-xylose concentrations in serum. Briefly, rats were fed with 1.5 mL of 5% D-xylose solution by gastric tubes. Blood samples were then collected, mixed with phloroglucinol color reagent and react in 100° C for 4 minutes. After cooling, the D-xylose concentrations in the serum were spectrophotometrically measured [63].

### D-latate test

The concentrations of D-latate in the plasma were measured by enzymatic-spectrophotometric method [64].

### Immunohistochemistry

Rats were euthanized and sacrificed on day 1, 3, 5, 7 after radiation. Four 2.5-cm sequential segments of proximal jejunum from the ligament of Treitz and the surrounding lymph nodes were obtained. Tissue samples were fixed in 10% neutral-buffered formalin for 12h, and then dehydrated and embedded with paraffin. Sections of 4 mm were used for hematoxylin-eosin staining (H&E staining). Paraffin-embedded sections were dewaxed, rehydrated and treated with 3% hydrogen peroxide. Following antigen retrieval, the sections were incubated with serum from the host for 30mins at room temperature to block nonspecific antigen-binding sites. Sections were washed and incubated with anti-PCNA (Abcam Cat# ab29, RRID:AB_303394), Signals were detected with the Envision kit (DAKO, Carpinteria, CA). Sections were counter-stained with hematoxylin. The number of positive cells in 5 crypts was scored in 100 crypts per section and reported as mean ± SD. Three rats were used in each group.

### Immunofluorescence

Rats were sacrificed 3 days after radiation and intestinal tissues were collected. Tissue samples were fixed in 10% neutral-buffered formalin for 12 h, dehydrated and embedded with paraffin. Following antigen retrieval, deparaffinized tissue sections were incubated with goat serum (Sigma-Aldrich) for 30min at room temperature to block nonspecific antigen-binding sites. For primary antibody, sections were incubated with anti-CD31 (Abcam Cat# ab28364, RRID:AB_726362), anti-Lgr5 (Thermo Fisher Scientific Cat# PA5-87974, RRID:AB_2804555) overnight at 4° C. For secondary antibody, sections were incubated with anti-rabbit Alexa Fluor 594 (Cell Signaling Technology Cat# 8889, RRID:AB_2716249) or anti-rabbit Alexa Fluor 488 (Abcam Cat# ab150077, RRID:AB_2630356) at 37° C for 1h. DAPI was used for nuclear staining. Positive signals were photographed under a fluorescent microscope (OLYMPUS BX43). Quantification of angiogenesis is represented as CD31^+^ area fraction analyzed by Image J (Image J, RRID:SCR_003070).

For *in vitro* study, IEC-6 cells were washed with PBS twice, fixed in 100% methanol, permeabilized by 0.3% Triton X-100, blocked in 10% goat serum on day 3 of experiment. Then cells were incubated with primary antibody anti-PCNA (Abcam Cat# ab29, RRID:AB_303394) overnight at 4° C. For secondary antibody, cells were incubated with anti-rabbit Alexa Fluor 594 (Cell Signaling Technology Cat# 8889, RRID:AB_2716249) at 37° C for 1 h. DAPI was used for nuclear staining. Positive signals were photographed under a fluorescent microscope (OLYMPUS BX43).

### TUNEL staining

Samples were collected from the intestines of irradiated rats and analyzed for apoptosis using deoxynucleotidyl transferase dUTP nick-end labeling (TUNEL) assay (In Situ Cell Death Detection Kit; Roche Applied Science, Indianapolis, IN). DAPI was used for nuclear staining. Each group contains at least 3 rats. The number of positive cells in 5 crypts was calculated in 100 crypts per section. The data was represented as mean ± SD.

### Flow cytometry

Rabbit anti-rat CD29 (BD Biosciences Cat# 555005, RRID:AB_395639), CD34 (Santa Cruz Biotechnology Cat# sc-74499, RRID:AB_1120394), CD44 (BD Biosciences Cat# 550974, RRID:AB_393987), CD45 (BD Biosciences Cat# 559135, RRID:AB_397196), CD105 (Abcam Cat# ab11414, RRID:AB_298019), CD90 (Santa Cruz Biotechnology Cat# sc-53456, RRID:AB_630308) were used to identify the BM-MSCs phenotypes. For intracellular staining, mesenteric lymph nodes were dissected into small pieces and incubated in RPMI 1640 medium containing collagenase type VIII at 200 U/ml (Sigma-Aldrich) for 50 minutes at 37° C while stirring. Supernatants containing cells were collected and the cells were washed and resuspended in complete RPMI 1640. The cells were then incubated with phorbol myristate acetate (50 ng/ml) and ionomycin (750 ng /ml) for 4 hours. Labeled cells were then analyzed on FACS. Monoclonal antibodies against CD4 (Thermo Fisher Scientific Cat# 11-0040-81, RRID:AB_953579) and Foxp3 (Thermo Fisher Scientific Cat# 12-5773-82, RRID:AB_465936) were used for flow cytometry.

For cell death detection, apoptosis of IEC-6 was measured by AnnexinV-FITC/PI Apoptosis Detection Kit (BD Bioscience, US) on 1, 3, 5, 7 days after radiation. Briefly, IEC-6 cells were seeded in 6-well plates (1×10^5^ cells/well). After radiation (10 Gy) and intervention at the indicated time, cells were washed with phosphate buffer saline twice, detached by trypsinization, centrifuged (200 g, 5mins), resuspended and stained with reagents from the Annexin V/propidium iodide (PI) Apoptosis Detection Kit for 10mins. The percentage of apoptotic cells was determined by flow cytometry.

### ELISA assay

VEGF, interleukin 6 (IL-6), basic fibroblast growth factor (bFGF), hepatocyte growth factor (HGF), interleukin-10 (IL-10), transforming growth factor-β (TGF-β1) in unconcentrated medium were measured by ELISA kit (R&D systems, Minneapolis, MN, US). Vascular cell adhesion molecule-1 (VCAM-1) were measured by ELISA kit (Abbexa, Cambridge, UK). Intestinal tissue samples and supernatant of IEC-6 were assayed by ELISA kit (R&D systems, Minneapolis, MN, US) for interleukin-1β (IL-1β), IL-10, tumor necrosis factor alpha (TNF-α).

### Quantification of NF-kB activity

The level of NF-κB p65 level was determined by using a commercially available ELISA kit (Active Motif, Carlsbad, CA) according to previous study [65]. Briefly, nuclear extract proteins of IEC-6 (5 μg) or intestines (10 μg) were prepared using the Nuclear Extract Kit (Active Motif, Carlsbad, CA) and incubated with an oligonucleotide containing the NF-κB consensus binding site (5′-GGGACTTTCC-3′) bound to a 96-well microtiter plate. The active form of NF-κB in the nuclear extract specifically binds to this consensus site. By using a primary antibody specific for the activated form of p65 of NF-κB and a secondary antibody conjugated to horseradish peroxidase, the activated form of p65 of NF-κB was evaluated by spectrophotometer (Thermo Scientific), which was read at 450 nm. All samples and standards were measured in duplicate.

### Cytokines agent

Recombinant rat VEGF 0.5 μg (Abcam, Cambridge, UK) and Recombinant rat IL-6 0.3 μg (Abcam, Cambridge, UK), were used to determine if VEGF and IL-6 have the same therapeutic effect as RG1-MSC-CM, both of which are equivalent to the content of 4ml RG1-MSC-CM in 4 days.

### Western blot

IEC-6 were lysed in radioimmunoprecipitation (RIPA) buffer on the ice for 10mins, and centrifuged at 10,000 r/min for 10mins. Protein concentrations were measured using BCA kits. The supernatant was separated on 10% SDS polyacrylamide electrophoresis gels and transferred to nitrocellulose membranes. After being blocked 5% non-fat milk (Sigma-Aldrich Corporation, St. Louis, Mo), the membranes were incubated with indicated primary antibody of HO-1 (Abcam Cat# ab13248, RRID:AB_2118663), p53 (Abcam Cat# ab26, RRID:AB_303198) at 4° C overnight and then incubated with peroxidase-conjugated secondary antibody (Cell Signaling Technology Cat# 7076, RRID:AB_330924) at 37° C for 1h. Antibody-antigen complexes on the membranes were detected using an ECL system (Amersham Life Sciences, Buckinghamshire, UK). A β-tubulin or GAPDH antibody at a 1:1,000 dilution was used as the control. Integrated density was measured by Image J.

### Quantitative real-time PCR assay

Total RNA was extracted from BM-MSCs using TRIzol reagent (ThermoFisher Scientific Inc., Carlsbad, CA) according to the manufacturer’s instructions. Quantitative PCR was carried out using SYBR® Premix ExTaqTM (Takara) in the LightCycler 480 (Roche Applied Science, Indianapolis, IN). The following primers were used:

VEGF Forward: 5′-AAATCCTGGAGCGTTCACTGTG-3′;

VEGF Reverse: 5′-AACGCGAGTCTGTGTTTTTGC-3′

IL-6 Forward: 5′-TCTCCACAAGCGCCTTGG -3′;

IL-6 Reverse: 5′-CTCAGGGCTGAGATGCCC-3′

IL-10 Forward: 5′-ACTGCTATGTTGCCTGCTCTT-3′;

IL-10 Reverse: 5′-TCATTCTTCACCTGCTCCACT-3′

The cycling conditions were 95° C for 30s followed by 40 cycles of 95° C for 5s and 60° C for 3s.

The relative mRNA values were normalized to the β-actin (a normalization control), gene control values and calculated using the 2−ΔΔCt method. Thereafter, data for transcript expression levels were expressed as fold difference relative to negative control cells.

### siRNA preparation and transfection

The sense and antisense strands of rat HO-1 siRNA were: 59-AAG CCA CAC AGC ACU AUG UAA dTdT-39 (sense) and 59-UUA CAU AGU GCU GUG UGG CUU dTdT-39 (antisense); Nonspecific siRNA (sense, 59-UUC UCC GAA CGU GUC ACG UdTdT-39; antisense, 59-ACG UGA CAC GUU CGG AGA AdTdT-39) was synthesized by Qiagen (Germantown, MD). The transfection process is based on instruction from Lifetechnologies. Briefly, BM-MSCs were trypsinized and seed in 6-well gelatin-coated plates (5 × 10^4^cells/well), 2 ml of growth medium without antibiotics was added so that cells will be 70%–80% confluent at the time of transfection. Oligofectamine reagent (Invitrogen) was used as the transfection agent, and cells were then incubated for 6 h. Next, fetal bovine serum/DMEM-F12 was added to reach a final concentration of 10% fetal bovine serum in the wells. After transfection, MSCs were stimulated by RG1.

### Statistical analysis

Data was analyzed using SPSS 17.0 software (SPSS, RRID:SCR_002865) and expressed as mean ± SD. The data were analyzed by using student t-test or one or two-way ANOVA followed by the Bonferroni post-hoc test in order to decide statistical significance. Kaplan-Meier method was applied to analyzed animal survival curves. A value of *P* < 0.05 was considered to be statistically significant.
